# Prevalence and determinants of symptoms of antenatal common mental disorders among women who had recently experienced an earthquake: a systematic review

**DOI:** 10.1186/s12888-018-1986-2

**Published:** 2019-01-28

**Authors:** Goma Kumari Khatri, Thach Duc Tran, Jane Fisher

**Affiliations:** School of Public Health and Preventive Medicine, Monash University, Level 4, 553 St Kilda Rd, Melbourne, Victoria 3004 Australia

**Keywords:** Antenatal, Earthquake, Common mental disorders, Women

## Abstract

**Background:**

Antenatal common mental disorders (CMDs) including anxiety, depressive, adjustment, and somatoform disorders are prevalent worldwide. There is emerging evidence that experiencing a natural disaster might increase the risk of antenatal CMDs. This study aimed to synthesise the evidence about the prevalence and determinants of clinically-significant symptoms of antenatal CMDs among women who had recently experienced an earthquake.

**Methods:**

This systematic review was conducted using the Preferred Reporting Items for Systematic Reviews and Meta-Analysis (PRISMA) guidelines. The search included both electronic and manual components. Five major databases were searched. A data extraction table was used to summarise study characteristics and findings. Two authors examined the quality of studies independently using a quality assessment tool. A narrative synthesis of the findings reported.

**Results:**

In total seven articles met inclusion criteria. Quality scores ranged from six to seven out of ten. All the studies were cross-sectional surveys and were conducted in high and middle-income countries. Sample sizes varied among studies. The prevalence of clinically-significant symptoms of antenatal CMD ranged from 4.6% (95% CI, 3.2; 6.5) experiencing ‘psychological stress’ in Japan to 40.8% (95% CI, 35.5; 46.4) ‘depression’ in China. While all studies were conducted in an earthquake context, only four examined some aspect of earthquake experiences as a risk factor for antenatal CMDs. In multivariable analyses, higher marital conflict, poor social support, multiparity, stresses of pregnancy and the personality characteristic of a negative coping style were identified as risks and a positive coping style as protective against antenatal CMDs.

**Conclusions:**

This systematic review found that women who have recently experienced an earthquake are at heightened risk of antenatal mental health problems. It indicates that in addition to the establishment of services for safe birth which is recognised in post-disaster management strategies, pregnancy mental health should be a priority. The review also revealed that there is no evidence available from the world’s low-income nations where natural disasters might have more profound impacts because local infrastructure is more fragile and where it is already established that women experience a higher burden of antenatal CMDs.

**Trial registration:**

PROSPERO-CRD42017056501.

## Introduction

Antenatal common mental disorders (CMDs) which include anxiety, depressive, adjustment, and somatoform disorders [1] are prevalent worldwide. In a systematic review conducted in 2004 which included 21 papers representing 13 high-income countries, Bennett et al. [2] reported that about 10% of women experienced ‘depression’ (although most studies had ascertained outcomes with symptom checklists and not diagnostic interviews), during pregnancy. The rate is much higher in low- and middle-income countries. Fisher et al. [3] reviewed all available papers published to 2010 from low- and lower-middle-income countries. The review found 13 studies but from only nine low- and lower-middle-income countries. The review reported a weighted mean prevalence of antenatal CMDs in these settings of 15.6% (95% CI, 15.4–15.9). The latest systematic review of antenatal ‘depression’ published in 2016 which included 51 studies published from 1998 to 2015 and representing 20 low- and middle-income countries. The study found that a pooled prevalence of ‘depression’ during pregnancy was 25.8% (95% CI, 22.8–29.0) [4]. This review reported significant heterogeneity and publication bias between studies [4]. This could be possible explanations for the much higher prevalence in this review than those of the previous study. Nevertheless, these reviews showed that the prevalence of antenatal CMDs is high worldwide.

Some determinants of antenatal CMDs in general circumstances have been established. Lancaster et al.’s [5] systematic review of ‘risk factors for depressive symptoms during pregnancy’ included 57 studies from the USA, Canada, Europe, Australia and New Zealand published from 1980 to 2008. They found in univariable comparisons that life stress, anxiety, a personal history of depression, lack of social support, unintended pregnancy, domestic violence, less education, lower income, smoking, being the single and poor quality of relationship were more common among with than those without antenatal depressive symptoms. When all were entered into multivariable analyses, life stress, lack of social support and domestic violence remained significantly more common among women with than without symptoms. Fisher et al.’s [3] systematic review from low- and lower-middle-income countries also reported that lack of support, socio-economic disadvantage, unintended pregnancy and intimate partner violence were risks and having more education, a permanent job, and a kind and trustworthy intimate partner protected against CMDs of women who were pregnant or had recently given birth [3].

There is limited empirical research about the links between experiences of a disaster and prevalence of antenatal CMDs. Harville et al. [6] conducted a systematic review of ‘disasters and perinatal health’ in 2011 and included ten papers reporting findings of the mental health of women who were pregnant or had recently given birth and had experienced a natural or a ‘technological’ disaster. The review found that exposure to a disaster can be stressful and that the mental health of women who are pregnant or in the postpartum period may have adverse consequences for the child’s development and that these might be greater than the direct effects of the disaster [6]. Ren et al. [7] reported similar findings in their systematic review of eight papers on ‘mental disorders of pregnant and postpartum women after earthquakes’. Some studies had examined factors other than the disaster as determinants of antenatal CMDs and found that poor social support and the poor family relationship may increase the risk of perinatal CMDs [7].

These two systematic reviews included some papers reporting experiences of earthquakes, other natural disaster and antenatal CMDs and indicated that earthquake might increase the risk of antenatal CMDs [6, 7]. However, to our knowledge, there is no systematic review of the prevalence and determinants of antenatal CMDs among women who recently experienced an earthquake. Antenatal CMDs among women is a global concern not only because of the burden and limits to participation in everyday affairs but also because of the risk to the neurocognitive development of the growing foetus and adverse pregnancy outcomes [8]. In addition, it is well-established that a history of antenatal CMDs is a risk factor for postnatal depression [9].

The aim of this study was to identify and synthesise the evidence available about the prevalence and determinants of clinically-significant symptoms of antenatal CMDs among women who have recently experienced an earthquake.

## Methods

We used the Preferred Reporting Items for Systematic Reviews and Meta-Analysis (PRISMA) guidelines [10]. The protocol was registered in PROSPERO-CRD42017056501 [11].

### Search strategy and data source

The search incorporated both electronic and manual components. The electronic databases: Psychi Info, Cochrane Library, PubMed, Scopus, Medline, Web of Science, CINAHL, ProQuest were searched. We used keywords, boolean operators and truncation to search for the relevant articles. The search terms were: (pregnan* or antenatal or antepartum or maternal or perinatal or prenatal or gestation or reproductive health or sexual health) AND (mental health problem* or common mental disorder* or depression or anxiety or psychosis or mental health status or mental health or mental disease* or mental well*being or mental health or post*traumatic stress disorder*) AND (disaster* or earthquake*). These search terms revised according to the specificities of each database.

The reference lists of articles that met inclusion criteria were searched manually to identify any further publications which had not been identified in the electronic searches.

### Eligibility criteria

Eligibility criteria were that papers had to report studies from any country that: 1) were about women who had experienced an earthquake in the prior five years, 2) ascertained and reported the prevalence of symptoms of antenatal CMDs using a standard method, 3) had been published in the English in the peer-reviewed literature to October 31st 2017.

### Data extraction

A data extraction table was used to summarise study characteristics and findings. We summarised the prevalence of clinically-significant symptoms of CMDs and extracted or derived confidence interval, odds ratio, relative risk, coefficients, and significance of determinants of CMDs.

### Quality assessment and analysis

Two authors (GKK and TDT) examined the quality of studies independently using a quality assessment tool designed by Greenhalgh [12] and modified by Fisher et al. [3] (Table 1). The authors discussed any discrepancies and reached a consensus. Quality assessment items included: “clear study aim, appropriate sample size (or justification), explicit inclusion and exclusion criteria, a measure of mental health standardized, a measure of mental health locally validated, response rate reported and losses were given, an adequate description of data, appropriate statistical analysis and appropriate informed consent.” These were scored one for meeting the criterion and zero for not meeting it, and the total potential score was ten.

**Table 1:** Methodological qualities of studies of symptoms of antenatal CMDs among women who had an earthquake experience (total score 10)

Study	The clear study aim	Appropriate justification for sample size	Representative sample (with justification)	Clear inclusion and exclusion criteria	A measure of mental health standardised	A measure of mental health locally validated	Response rate reported and losses given	Adequate description of data	Appropriate statistical analysis	Appropriate informed consent procedure	Total score
Chang Hseuh-ling, et al., 2002 [18]	1	0	0	1	1	0	1	1	1	1	7
Hibino Yuri, et al., 2009 [13]	1	0	0	1	1	0	1	1	1	1	7
Lau Ying, et al., 2011 [17]	1	0	0	1	1	0	1	1	0	1	6
Qu Zhiyong, et al., 2012 [15]	1	0	0	0	1	1	0	1	1	1	6
Dong Xuehan, et al., 2013 [16]	1	0	0	0	1	1	1	1	1	1	7
Ren et al., 2015 [19]	1	0	0	1	1	0	1	1	1	1	7
Watanabe et al., 2016 [14]	1	0	0	1	1	0	1	1	1	1	7

## Results

**Fig. 1:**
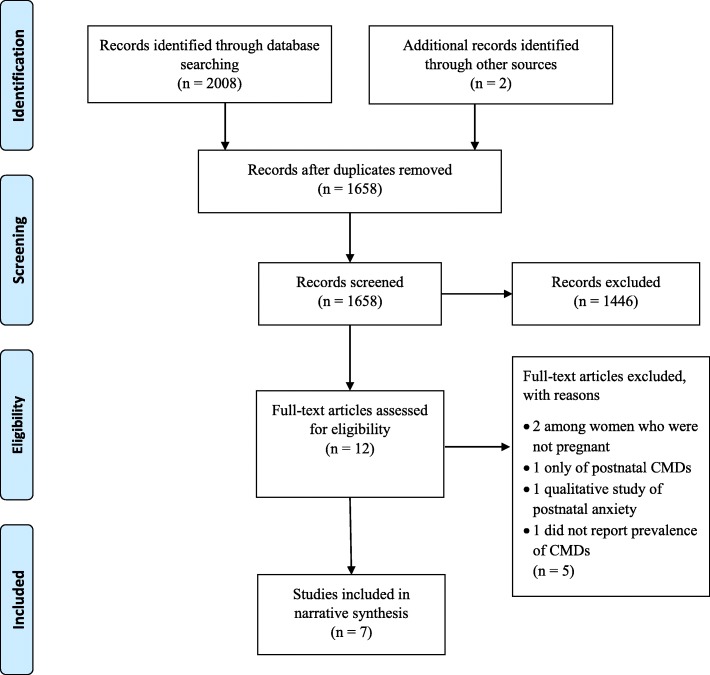
Flow diagram of selection of eligible papers

The steps to select eligible papers based on the eligibility criteria are reported in Fig. 1. In total, seven articles met inclusion criteria. Quality scores ranged from six to seven out of ten (Table 1). As the studies were heterogeneous in methodology, we were unable to undertake a meta-analysis and therefore completed a narrative synthesis of the findings.

### Study characteristics

All the studies were conducted in Japan, Taiwan, or China. All were cross-sectional surveys. In total, data were contributed by 2209 women who had experienced an earthquake and 7381 women in comparison conditions who had not. Sample sizes varied among studies. The smallest was Hibino et al.’s investigation of 99 [13] women and the largest, Watanabe et al.’s [14] of 670 women who experienced an earthquake and 6803 as a comparison group, both in Japan. None of the seven studies justified the sample size or sought to establish representativeness (Table 1). While Qu et al. [15] described their study as having ‘randomised sampling’ and Dong et al. [16] and Lau et al. [17] mentioned that they had ‘non-probabilistic convenience sampling’ none reported anything about the recruitment strategies.

Six studies [13, 15–19] recruited participants from antenatal clinics in hospitals. One study [14] recruited participants from antenatal clinics or ‘local government offices issuing [the] pregnancy journal, Mother-Child Health Handbook’. This is an official booklet which is provided free to all expectant mothers when they register to receive municipal services for pregnancy, birth and childcare in Japan [14].

Although all studies were conducted in an earthquake context, the magnitude of earthquakes, the distance between the study site and the epicentre, and timing of experience of the earthquakes varied among studies (Table 2). The magnitude of earthquakes was from 6.9 [13] to 9.0 [14] on the Richter Scale. Four studies [14–17] were conducted in areas which had experienced earthquakes rated at least eight on the Richter Scale. The distance of the study setting from the epicentre varied from zero kilometres [13, 19] to 90 km [17].

**Table 2:** Studies examining CMDs during pregnancy who experienced an earthquake

Study/type	Setting	Distance between study site and the earthquake epicentre	Magnitude of earthquake (Richter scale)/epicentre	When had experienced an earthquake	Sample for those who experienced an earthquake	Sample of comparison group	Gestational age at the time of study (trimester)	Assessment instrument and cut off score	Locally validated instrument among pregnant women	Prevalence of CMDs (%) (95% CI): those who experienced an earthquake	Prevalence of CMDs (%) (95% CI): comparison group	*P*-value for difference in prevalence
Middle economy												
Lau Ying, et al., 2011 [17], Cross-sectional	Four hospitals, Chengdu, Sichuan China	90 km	8.0, Wenchuan county, Sichuan province	During pregnancy	578	578	2	EPDS ≥14	No	7.1 (5.1; 9.5);	9.2 (6.9; 11.8)	–
Qu Zhiyong, et al., 2012 [15], Cross-sectional	Mianzhu People’s hospital and Mianzhu Maternal and Child Health hospital, China	30 km	8.0, Wenchuan county, Sichuan province	18 months before pregnancy	311	No comparison group	1, 2, 3	EPDS ≥10 IES-*R* ≥ 2	Yes	40.8 (35.5; 46.4)	–	
									No	12.2 (9.0; 16.4)	–	
Dong Xuehan, et al., 2013 [16], Cross-sectional	Mianzhu People’s hospital, Mianzhu Maternal and Child Health hospital and Gaobeidian County Hospital, China	30 km	8.0, Wenchuan county, Sichuan province	4 years before pregnancy	252	268	2	EPDS ≥10	Yes	34.5 (28.9; 40.6);	39.6 (33.9; 45.5)	*P* = 0.236
Ren et al., 2015 [19], Cross-sectional	3 hospitals of Ya’an City, China	0 km	7.0, Lushan county, Ya’an city	During pregnancy	128	No comparison group	2	EPDS ≥14	No	35.2 (26.9; 44.1)		
High economies												
Chang Hseuh-ling, et al., 2002 [18], Cross-sectional	Antenatal clinic of Pu-Li Christian Hospital, Taiwan	a few km away (exact km not reported)	7.3, central Taiwan	During or 6 to 12 months before pregnancy	171	No comparison group	Not reported	CHQ-12 > 3	No	29.2 (22.5; 36.7)	–	
Hibino Yuri, et al., 2009 [13], Cross-sectional	Four hospitals, Noto area Japan	0 km	6.9, Noto Peninsula	During or about 3 months before pregnancy	99	No comparison group	2, 3	EPDS ≥10	No	13.1 (7.2; 21.4)	–	
Watanabe et al., 2016 [14], Cross-sectional	14 units centre from 7 municipalities in Japan	near to epicentre (km not reported)	9.0, near Miyagi	During pregnancy	670	6803	Not reported	K6 ≥ 13	No	4.6 (3.2; 6.5)	3.1 (2.7; 3.6)	–

Note: *CI* Confidence Interval, *km* kilo meter, *CMD* Common Mental Disorder, *EPDS* Edinburgh Postnatal Depression Scale, *IES-R* Impact of Event Scale-Revised, *CHQ* Chinese Health Questionnaire, *K6* Kessler −6

Three studies [14, 17, 19] recruited and interviewed pregnant women who were pregnant at the time of the earthquake. Two studies were conducted among pregnant women who experienced earthquakes 18 months [15] and four years [16] before pregnancy. The other two studies [13, 18] interviewed pregnant women who experienced earthquakes either a few months before or during pregnancy.

Three studies [14, 16, 17] had a comparison group. Dong et al. [16] and Watanabe et al. [14] recruited pregnant women from a non-earthquake affected area as a comparison group. Dong et al. [16] reported that they recruited pregnant women from Gaobeidian county, Heibei province, non-earthquake affected area as a comparison group following the same recruitment method of the study group. However, they did not report how they selected the comparison group. Watanabe et al. [14] reported that they selected a comparison group (but not how) from an area which had ‘no or little direct effects’ due to the earthquake in which they followed the same recruitment method as used for the earthquake experienced group. Lau et al. [17] compared a pre-earthquake and a post-earthquake group which had been recruited from the same area using the same methods but did not report inclusion criteria. Two of these studies [14, 17] reported that the characteristics of the study group differed significantly from those of the comparison group, including in family income, educational level [14], and employment status [17] which might have influenced the outcomes. Dong et al. [16] reported number and percentage of data but did not report any significant difference between study and comparison data.

All studies used a standardised screening tool to measure CMD. Five studies [13, 15–17, 19] used the Edinburgh Postnatal Depression Scale (EPDS) to measure pregnancy mental health, but these studies used different cutoff scores to indicate clinically significant symptoms: ≥ 10 for three [13, 15, 16] and ≥ 14 for two [17, 19] studies. In only two [15, 16] of these studies had a cutoff score been established in a local formal validation among pregnant women. One study used scores of > 3 on the Chinese Health Questionnaire (CHQ)-12 [18] and another used ≥13 scores on Kessler (K)-6 [14]. Neither of these cutoff scores had been established in local validations among pregnant women but had been among the general population [14, 18].

### Prevalence of symptoms of antenatal CMDs

The prevalence of clinically-significant symptoms of antenatal CMD ranged from 4.6% (95% CI, 3.2; 6.5) experiencing ‘psychological stress’ in Japan [14] to 40.8% (95% CI, 35.5; 46.4) ‘depression’ in China [15] (Table 2).

Of four studies in China, two reported the prevalence of depressive symptoms among women who had experienced the earthquake during pregnancy: 7.1% (95% CI, 5.1; 9.5) [17] and 35.2% (95% CI, 26.9; 44.1) [19] using the same EPDS cut off score ≥ 14. The other two studies reported the prevalence of depressive symptoms among women who had experienced earthquakes 18 months (40.8% (95% CI, 35.5; 46.4)) [15] and 4 years (34.5% (95% CI, 28.9; 40.6)) [16] (EPDS cut off score ≥ 10) before pregnancy. Qu et al. [15]‘s was the only study which investigated trauma reactions among pregnant women in an earthquake context and reported the prevalence of post-traumatic stress disorder (PTSD): 12.2% (95% CI, 9.0; 16.4) using ≥2 cut-off scores of Impact of Event Scale-Revised (IES-R).

Among the studies in Japan, Hibino et al. [13] found that 13.1% (95% CI, 7.2; 21.4) of pregnant women had depressive symptoms (≥10 EPDS cut off score). Watanabe et al. [14] reported that 4.6% (95% CI, 3.2; 6.5) pregnant women had ‘psychological stress’ (≥13 K6 scores). Similarly, the study in Taiwan [18] reported that 29.2% (95% CI, 22.5; 36.7) pregnant women experienced ‘minor psychiatric morbidity’ (> 3 CHQ-12 score).

### Determinants for antenatal CMDs

While all studies examined some determinants, none included all potential risk and protective factors for pregnancy mental health problems [3, 5] (Table 3). One study compared factors associated with differences in the prevalence between exposed and non-exposed groups, but they did not report the findings of these factors as determinants of CMDs [14]. Two studies [13, 18] conducted bivariate analyses only, and the other four studies [15–17, 19] conducted multivariate analyses to report potential determinants of antenatal CMDs. Studies controlled some common but some different covariates in the multivariate analyses. The key findings of the factors are grouped and described in sub-sections below, and details are presented in Table 3.

**Table 3:** Determinants of clinically-significant symptoms of antenatal CMDs among women who experienced an earthquake recently

Determinants	Association with clinically-significant symptoms of antenatal CMDs	Sample size and covariates controlled for
Earthquake experiences		
Wounded themselves	Relative Risk, 1.28 (95% CI, 0.51; 3.23, *p* = 0.61) [18]	*n* = 171; Covariates: none [18]
Wounded relatives	With EPDS total score (adjusted regression coefficient 0.221, *p* = 0.002) [19]	*n* = 128; Covariates: number of children, subjective support, negative coping styles and positive coping styles [19]
Dead relatives	Mean EPDS score: yes (21.67 ± 2.51); no (11.00 ± 5.53 (*p* = 0.001) [19]	*n* = 128; Covariates: none [19]
	Relative Risk, 3.42 (95% CI, 2.71; 4.32, *p* = 0.003) [18]	*n* = 171; Covariates: none [18]
Starvation during pregnancy	Relative Risk, 2.34 (95% CI, 1.59; 3.44, *p* < 0.001) [18]	*n* = 171; Covariates: none [18]
The intensity of an earthquake ((less than 6 upper/6 upper seismic intensity on Japanese scale)	Correlation ratio with EPDS total score (ɳ=0.16, p < 0.01) [13]	*n*^a^; Covariates: none [13]
Composite earthquake experiences (12-item scale)	≥2 IES-R score: (aOR, 1.80, 95% CI, 1.43; 2.26; *p* < 0.001) [15]	*n* = 311; Covariates: age, education and stresses of pregnancy [15]
Displacement due to earthquake	Relative Risk, 1.01 (95% CI, 0.73; 1.42, *p* = 0.93) [18]	*n* = 171; Covariates: none [18]
House damage	Correlation ratio with EPDS total score (ɳ=0.04, *p* > 0.05) [13]	*n*^a^; Covariates: none [13]
Evacuation	Correlation ratio with EPDS total score (ɳ=0.08, > 0.05) [13]	*n*^a^; Covariates: none [13]
Support		
Subjective support	With EPDS total score (adjusted regression coefficient = − 0.249, *p* = 0.001) [19]	*n* = 128; Covariates: number of children, wounded relatives, negative coping styles and positive coping styles [19]
Objective support	Correlation coefficient with EPDS total score (*r* = − 0.324; *p* = 0.01) [19]	*n* = 128; Covariates: none [19]
Support use	Correlation coefficient with EPDS total score (*r* = − 0.320; *p* = 0.01) [19]	*n* = 128; Covariates: none [19]
Perceived availability of functional social support	≥14 EPDS score: Poor social support (aOR, 3.07, 95% CI, 1.21; 7.78, *p* < 0.05 [17]	*n* = 578; Covariates: age, education, duration of living in a study setting, employment status of respondents, a main financial supporter of the family, types of residence, number of babies and marital conflict [17]
Support from parents	≥10 EPDS score: compared to higher support, lower (aOR, 0.56, 95% CI, 0.06; 5.13, *p* = 0.603), medium (aOR, 1.34, 95% CI, 0.49; 3.67, *p* = 0.564) [16]	*n* = 520; Covariates: age, education, monthly family income, residence, employment, maternal BMI, pregnancy intention, gestational age, parity, sleep quality, smoking, alcohol history, husband is migrant worker, social support from husband, parents-in-law, stresses of pregnancy, marital satisfaction, thoughts and feelings regarding the marriage and one’s spouse, agreement on relationship matters and life satisfaction [16]
Support from parents-in-law	≥10 EPDS score: compared to higher support, lower (aOR, 0.74, 95% CI, 0.24; 2.23, *p* = 0.588), medium (aOR, 0.51, 95% CI, 0.24; 1.08, *p* = 0.076) [16]	*n* = 520; Covariates: age, education, monthly family income, residence, employment, maternal BMI, pregnancy intention, gestational age, parity, sleep quality, smoking, alcohol history, husband is migrant worker, social support from husband, parents, stresses of pregnancy, marital satisfaction, thoughts and feelings regarding the marriage and one’s spouse, agreement on relationship matters and life satisfaction [16]
Socio-demographic factors		
Age	≥ 14 EPDS score: compared to > 25 years, ≤25 years (aOR, 1.79; 95% CI, 0.84; 3.85; *p* > 0.05) [17]	*n* = 578; Covariates: education, duration of living in the study setting, employment status of respondents, a main financial supporter of the family, types of residence, number of babies, marital conflict and social support [17]
	≥2 IES-R score: compared to ≥30 years, younger age (18–24 years) (aOR, 0.10, 95% CI, 0.03; 0.31, *p* < 0.001), aged 25 to 29 years (aOR, 0.35, 95% CI, 0.09; 1.34, *p* = 0.125) [15]	*n* = 311; Covariates: education, stress of pregnancy and earthquake experiences [15]
	≥10 EPDS score: compared to ≥30 years, younger age (18–24 years) (aOR, 0.42, 95% CI, 0.20; 0.90, *p* = 0.025), aged 25 to 29 years (aOR, 0.56, 95% CI, 0.22; 1.39, *p* = 0.210) [15]	*n* = 311; Covariates: education, pregnancy intention, sleep quality, family relationship and stresses of pregnancy [15]
	≥10 EPDS score: compared to ≥30 years, younger age (18–24 years) (aOR, 0.96, 95% CI, 0.41; 2.27, *p* = 0.927), aged 25–29 years (aOR, 0.60, 95% CI, 0.25; 1.43, *p* = 0.246) [16]	*n* = 520; Covariates: education, monthly family income, residence, employment, maternal BMI, pregnancy intention, gestational age, parity, sleep quality, smoking, alcohol history, husband is migrant worker, social support from husband, parents, parents-in-law, stresses of pregnancy, marital satisfaction, thoughts and feelings regarding the marriage and one’s spouse, agreement on relationship matters and life satisfaction [16]
	Correlation coefficient with EPDS total score (*r* = − 0.014, *p* > 0.05) [19]	*n* = 128; Covariates: none [19]
	> 3 CHQ score: 28.04 ± 5.5, < 3 CHQ score: 27.16 ± 4.7, *p* = 0.32 [18]	*n* = 171; Covariates: none [18]
	Correlation coefficient with EPDS total score (*r* = − 0.12, *p* > 0.05) [13]	*n*^a^; Covariates: none [13]
Maternal body weight (kg)	> 3 CHQ score: 54.15 ± 8.7, < 3 CHQ score: 53.67 ± 8.7, *p* = 0.23 [18]	*n* = 171; Covariates: none [18]
Maternal height (cm)	> 3 CHQ score: 158.1 ± 4.9, < 3 CHQ score: 157.6 ± 5.3, *p* = 0.25 [18]	*n* = 171; Covariates: none [18]
Maternal body mass index (BMI)	≥10 EPDS score: < 23/≥23 (aOR, 1.89, 95% CI, 0.99; 3.64, *p* = 0.056) [16]	*n* = 520; Covariates: age, education, monthly family income, residence, employment, pregnancy intention, gestational age, parity, sleep quality, smoking, alcohol history, husband is migrant worker, social support from husband, parents, parents-in-law, stresses of pregnancy, marital satisfaction, thoughts and feelings regarding the marriage and one’s spouse, agreement on relationship matters and life satisfaction [16]
Education	≥14 EPDS score: compared to Tertiary, ≤secondary (aOR, 1.27; 95% CI, 0.58; 5.79; *p* > 0.05) [17]	*n* = 578; Covariates: age, duration of living in the study setting, employment status of respondents, a main financial supporter of the family, types of residence, number of babies, marital conflict and social support [17]
	≥2 IES-R score: compared to college or above, middle or lower education (aOR, 1.52, 95% CI, 0.23; 9.92, *p* = 0.660); high school (aOR, 0.27, 95% CI, 0.03; 2.11, *p* = 0.210) [15]	*n* = 311; Covariates: age, stresses of pregnancy and earthquake experiences [15]
	≥10 EPDS score: compared to college or above, middle or lower education (aOR, 0.56, 95% CI, 0.24; 1.32,*p* = 0.186); high school (aOR, 1.29, 95% CI, 0.55; 3.02, *p* = 0.565) [16]	*n* = 520; Covariates: age, monthly family income, residence, employment, maternal BMI, pregnancy intention, gestational age, parity, sleep quality, smoking, alcohol history, husband is migrant worker, social support from husband, parents, parents-in-law, stresses of pregnancy, marital satisfaction, thoughts and feelings regarding the marriage and one’s spouse, agreement on relationship matters and life satisfaction [16]
	EPDS scores of ≤ primary school (13.29 ± 5.53), high school (11.86 ± 5.76), junior college (10.21 ± 5.89), ≥ bachelor’s degree (8.93 ± 4.46) (*p* = 0.105) [19]	*n* = 128; Covariates: none [19]
Occupation	EPDS scores of farmers (13.07 ± 5.93), others (9.65 ± 5.03) (*p* = 0.001) [19]	*n* = 128; Covariates: none [19]
Employment	≥14 EPDS score: compared to full-time employed, part-time/unemployment (aOR, 1.00; 95% CI, 0.46; 2.17; *p* > 0.05) [17]	*n* = 578; Covariates: age, education, duration of living in a study setting, a main financial supporter of the family, types of residence, number of babies, marital conflict and social support [17]
	≥10 EPDS score: compared to full-time job, unemployed (aOR, 0.54, 95% CI, 0.21; 1.39, *p* = 0.201), part-time job (aOR, 2.97, 95% CI, 0.58; 13.85, *p* = 196) [16]	*n* = 520; Covariates: age, education, monthly family income, residence, maternal BMI, pregnancy intention, gestational age, parity, sleep quality, smoking, alcohol history, husband is migrant worker, social support from husband, parents, parents-in-law, stresses of pregnancy, marital satisfaction, thoughts and feelings regarding the marriage and one’s spouse, agreement on relationship matters and life satisfaction [16]
Partner’s employment status	≥14 EPDS score: compared to full-time employed, part-time/unemployment (OR, 1.40, 95% CI, 0.31; 6.22, *p* > 0.05) [17]	*n* = 578; Covariates: none [17]
Monthly individual total income	≥14 EPDS score: compared to >RMB $2000, ≤RMB $2000 (OR, 1.52, 95% CI, 0.78; 2.94, *p* > 0.05) [17]	*n* = 578; Covariates: none [17]
Monthly family income	≥10 EPDS score: compared to ≥802 USD, < 160 USD (aOR, 0.98, 95% CI, 0.31; 3.04, *p* = 0.965), 160–320 USD (aOR, 1.72, 95% CI, 0.76; 3.89, *p* = 0.194), 321–801 USD (aOR, 0.80, 95% CI, 0.39; 1.64, *p* = 0.540) [16]	*n* = 520; Covariates: age, education, residence, employment, maternal BMI, pregnancy intention, gestational age, parity, sleep quality, smoking, alcohol history, husband is migrant worker, social support from husband, parents, parents-in-law, stresses of pregnancy, marital satisfaction, thoughts and feelings regarding the marriage and one’s spouse, agreement on relationship matters and life satisfaction [16]
	EPDS score of < 5000 (12.26 ± 5.99), 5000 to 9999 (13.0 ± 7.05), 10,000 to 19,999 (9.29 ± 4.98), 20,000 to 49,999 (10.00 ± 3.91), ≥50,000 (9.18 ± 4.42) (*p* = 0.80) [19]	*n* = 128; Covariates: none [19]
Main finance supporter in the family	≥14 EPDS score: compared to couple sharing, one partner only (aOR, 2.19, 95% CI, 1.00; 4.80, *p* > 0.05) [17]	*n* = 578; Covariates: age, education, duration of living in a study setting, employment status of respondents, types of residence, number of babies, marital conflict and social support [17]
Type of residence	≥14 EPDS score: compared to private house, in public house (aOR, 1.20, 95% CI, 0.56; 2.57, *p* > 0.05) [17]	*n* = 578; Covariates: age, education, duration of living in a study setting, employment status of respondents, a main financial supporter of the family, number of babies, marital conflict and social support [17]
	≥10 EPDS score: Village/City (aOR, 1.57, 95% CI, 0.69; 3.59, *p* = 0.285) [16]	*n* = 520; Covariates: age, education, monthly family income, employment, maternal BMI, pregnancy intention, gestational age, parity, sleep quality, smoking, alcohol history, husband is migrant worker, social support from husband, parents, parents-in-law, stresses of pregnancy, marital satisfaction, thoughts and feelings regarding the marriage and one’s spouse, agreement on relationship matters and life satisfaction [16]
Duration of stay in the study area	≥14 EPDS score: compared to > 1 year, < 1 year (aOR, 3.58; 95% CI, 1.16; 11.01, *p* < 0.05) [17]	*n* = 578; Covariates: age, education, employment status of respondents, a main financial supporter of the family, types of residence, number of babies, marital conflict and social support [17]
Sleep quality	≥10 EPDS score: compared to very good, poor (aOR, 1.47, 95% CI, 0.48; 4.54, *p* = 0.504); fair (aOR, 1.57, 95% CI, 0.78; 3.16, *p* = 0.203); good (aOR, 0.74, 95% CI, 0.33; 1.62, *p* = 0.445) [15]	*n* = 311; Covariates: age, education, pregnancy intention, family relationship and stresses of pregnancy [15]
	≥10 EPDS score: compared to good, poor (aOR, 2.38, 95% CI, 0.95; 5.99, *p* = 0.64), fair (aOR, 1.58, 95% CI, 0.89; 2.81, *p* = 0.20) [16]	*n* = 520; Covariates: age, education, monthly family income, residence, employment, maternal BMI, pregnancy intention, gestational age, parity, smoking, alcohol history, husband is migrant worker, social support from husband, parents, parents-in-law, stresses of pregnancy, marital satisfaction, thoughts and feelings regarding the marriage and one’s spouse, agreement on relationship matters and life satisfaction [16]
Smoking history	≥10 EPDS score: no/yes (aOR, 0.54, 95% CI, 0.07; 4.33, *p* = 0.560) [16]	*n* = 520; Covariates: age, education, monthly family income, residence, employment, maternal BMI, pregnancy intention, gestational age, parity, sleep quality, alcohol history, husband is migrant worker, social support from husband, parents, parents-in-law, stresses of pregnancy, marital satisfaction, thoughts and feelings regarding the marriage and one’s spouse, agreement on relationship matters and life satisfaction [16]
Alcohol use history	≥10 EPDS score: no/yes (aOR, 1.35, 95% CI, 0.42; 4.31, *p* = 0.615) [16]	*n* = 520; Covariates: age, education, monthly family income, residence, employment, maternal BMI, pregnancy intention, gestational age, parity, sleep quality, smoking history, husband is migrant worker, social support from husband, parents, parents-in-law, stresses of pregnancy, marital satisfaction, thoughts and feelings regarding the marriage and one’s spouse, agreement on relationship matters and life satisfaction [16]
Intimate partner relationship and support		
Length of marriage	≥14 EPDS score: compared to > 1 year, < 1 year (OR, 1.44, 95% CI, 0.72; 2.86, *p* > 0.05) [17]	*N* = 578; Covariates: none [17]
Husband is a migrant worker	≥10 EPDS score: no/yes (aOR, 1.08, 95% CI, 0.64; 1.83, *p* = 0.781) [16]	*n* = 520; Covariates: age, education, monthly family income, residence, employment, maternal BMI, pregnancy intention, gestational age, parity, sleep quality, smoking, alcohol history, social support from husband, parents, parents-in-law, stresses of pregnancy, marital satisfaction, thoughts and feelings regarding the marriage and one’s spouse, agreement on relationship matters and life satisfaction [16]
Marital satisfaction	≥10 EPDS score: with total score (aOR, 0.97, 95% CI, 0.90; 1.05, *p* = 0.434) [16]	*n* = 520; Covariates: age, education, monthly family income, residence, employment, maternal BMI, pregnancy intention, gestational age, parity, sleep quality, smoking, alcohol history, husband is migrant worker, social support from husband, parents, parents-in-law, stresses of pregnancy, thoughts and feelings regarding the marriage and one’s spouse, agreement on relationship matters and life satisfaction [16]
‘Thoughts and feelings regarding the marriage and one’s spouse’	≥10 EPDS score: with total score (aOR, 0.99, 95% CI, 0.97; 1.02, *p* = 0.776) [16]	*n* = 520; Covariates: age, education, monthly family income, residence, employment, maternal BMI, pregnancy intention, gestational age, parity, sleep quality, smoking, alcohol history, husband is migrant worker, social support from husband, parents, parents-in-law, stresses of pregnancy, marital satisfaction, agreement on relationship matters and life satisfaction [16]
‘Agreement on relationship matters’	≥10 EPDS score: with total score (aOR, 0.99, 95% CI, 0.96; 1.02, *p* = 0.401) [16]	*n* = 520; Covariates: age, education, monthly family income, residence, employment, maternal BMI, pregnancy intention, gestational age, parity, sleep quality, smoking, alcohol history, husband is migrant worker, social support from husband, parents, parents-in-law, stresses of pregnancy, marital satisfaction, thoughts and feelings regarding the marriage and one’s spouse and life satisfaction [16]
Quality of marital and family relationship	≥10 EPDS score: with total score (aOR, 0.84, 95% CI, 0.73; 0.98, *p* = 0.022) [15]	*n* = 311; Covariates: age, education, pregnancy intention, sleep quality and stresses of pregnancy [15]
Marital conflict ((The Dyadic Adjustment Scale Total Score)	≥14 EPDS score: higher conflict (aOR, 3.60, 95% CI, 1.76; 7.20, *p* < 0.01) [17]	*n* = 578; Covariates: age, education, duration of living in a study setting, employment status of respondents, a main financial supporter of the family, types of residence, number of babies and social support [17]
Support from husband	≥10 EPDS score: compared higher support, lower (aOR, 1.75, 95% CI, 0.16; 19.28, *p* = 0.646) medium (aOR, 3.57, 95% CI, 1.36; 9.38, *p* = 0.010) [16]	*n* = 520; Covariates: age, education, monthly family income, residence, employment, maternal BMI, pregnancy intention, gestational age, parity, sleep quality, smoking, alcohol history, husband is migrant worker, social support from parents, parents-in-law, stresses of pregnancy, marital satisfaction, thoughts and feelings regarding the marriage and one’s spouse, agreement on relationship matters and life satisfaction [16]
Reproductive factors		
Gestational age	≥10 EPDS score: < 12 weeks = 8 (47.1%); 13–28 weeks = 38 (31.7%); > 28 weeks = 78 (47.3%) (*p* = 0.025) [15]	*n* = 311; Covariates: none [15]
	≥10 EPDS score: 13–28 weeks/> 28 weeks (aOR, 1.06, 95% CI, 0.56; 2.01, *p* = 0.857) [16]	*n* = 520; Covariates: age, education, monthly family income, residence, employment, maternal BMI, pregnancy intention, parity, sleep quality, smoking, alcohol history, husband is migrant worker, social support from husband, parents, parents-in-law, stresses of pregnancy, marital satisfaction, thoughts and feelings regarding the marriage and one’s spouse, agreement on relationship matters and life satisfaction [16]
	Correlation coefficient with EPDS total score (*r* = 0.181, *p* < 0.05) [19] Mean EPDS score of > 28 weeks (11.75 ± 5.52); < 28 weeks (8.96 ± 6.14) (*p* = 0.033) [19]	*n* = 128; Covariates: none [19]
	Correlation ratio with EPDS total score (ɳ=0.21, *p* = < 0.05) [13]	*n*^a^; Covariates: none [13]
Pregnancy intention	≥14 EPDS score: compared to planned, unplanned (OR, 1.63, 95% CI, 0.80; 3.29, *p* > 0.05) [17]	*n* = 578; Covariates: none [17]
	≥10 EPDS score: compared to planned pregnancy, unplanned (aOR, 1.65, 95% CI, 0.96; 2.85, *p* = 0.070) [15]	*n* = 311; Covariates: age, education, sleep quality, family relationship and stresses of pregnancy [15]
	≥10 EPDS score: planned/unplanned (aOR, 0.92, 95% CI, 0.54; 1.55, *p* = 0.743) [16]	*n* = 520; Covariates: age, education, monthly family income, residence, employment, maternal BMI, gestational age, parity, sleep quality, smoking, alcohol history, husband is migrant worker, social support from husband, parents, parents-in-law, stresses of pregnancy, marital satisfaction, thoughts and feelings regarding the marriage and one’s spouse, agreement on relationship matters and life satisfaction [16]
Parity/number of children	≥14 EPDS score: compared to nulliparous, multiparous (aOR, 2.47, 95% CI, 1.18; 5.17; *p* < 0.05) [17]	*n* = 578; Covariates: age, education, duration of living in a study setting, employment status of respondents, a main financial supporter of the family, types of residence, marital conflict and social support [17]
	≥2 IES-R score: Primi gravida = 5(3.9%); others = 33 (18.0%) (*p* < 0.001) [15]	*n* = 311; Covariates: none [15]
	≥10 EPDS score: Primipara/Others (aOR, 0.54, 95% CI, 0.29; 1.01, *p* = 0.055) [16]	*n* = 520; Covariates: age, education, monthly family income, residence, employment, maternal BMI, pregnancy intention, gestational age, sleep quality, smoking, alcohol history, husband is migrant worker, social support from husband, parents, parents-in-law, stresses of pregnancy, marital satisfaction, thoughts and feelings regarding the marriage and one’s spouse, agreement on relationship matters and life satisfaction [16]
	with EPDS total score: number of children (adjusted regression coefficient = 0.262, *p* < 0.001) [19]	*n* = 128; Covariates: wounded relatives, subjective support and negative coping styles [19]
	Gravida > 3 CHQ score: 2.30 ± 0.95, < 3 CHQ score: 2.11 ± 1.10, *p* = 0.46 [18] Parity > 3 CHQ score: 0.97 ± 0.79, < 3 CHQ score: 0.93 ± 0.97, *p* = 0.53 [18]	*n* = 171; Covariates: none [18]
	Correlation ratio with EPDS total score: parity (ɳ=0.40, *p* < 0.01) [13]	*n*^a^; Covariates: none [13]
Perceived pressure of pregnancy	≥2 IES-R score: total score of stress scale (aOR, 1.19, 95% CI, 1.07; 1.32; *p* = 0.001) [15]	*n* = 311; Covariates: age, education earthquake experiences [15]
	≥10 EPDS score: total score of stress scale (aOR, 1.19, 95% CI, 1.12; 1.27; p < 0.001) [15]	*n* = 311; Covariates: age, education, pregnancy intention, sleep quality and family relationship [15]
	≥10 EPDS score: total score of stress scale (aOR, 4.55, 95% CI, 2.36; 8.77, p < 0.001) [16]	*n* = 520; Covariates: age, education, monthly family income, residence, employment, maternal BMI, pregnancy intention, gestational age, parity, sleep quality, smoking, alcohol history, husband is migrant worker, social support from husband, parents, parents-in-law, marital satisfaction, thoughts and feelings regarding the marriage and one’s spouse, agreement on relationship matters and life satisfaction [16]
Personality factors		
Positive coping	With EPDS total score: (adjusted regression coefficient = − 0.193, *p* = 0.006) [19]	*n* = 128; Covariates: number of children, wounded relatives, subjective support and negative coping styles [19]
Negative coping	With EPDS total score: (adjusted regression coefficient = 0.276, *p* < 0.001) [19]	*n* = 128; Covariates: number of children, wounded relatives, subjective support and positive coping styles [19]
Life satisfaction	≥10 EPDS score: with total score (aOR, 0.97, 95% CI, 0.93; 1.02, *p* = 0.267) [16]	*n* = 520; Covariates: age, education, monthly family income, residence, employment, maternal BMI, pregnancy intention, gestational age, parity, sleep quality, smoking, alcohol history, husband is migrant worker, social support from husband, parents, parents-in-law, stresses of pregnancy, marital satisfaction, thoughts and feelings regarding the marriage and one’s spouse and agreement on relationship matters [16]
‘Negative attitude to the influence of earthquake on pregnancy’	Relative Risk, 1.28 (95% CI, 0.89; 1.85, *p* = 0.19) [18]	*n* = 171; Covariates: none [18]
‘Existing anxiety about an earthquake’	Correlation ratio with EPDS total score (ɳ=0.50, *p* < 0.01) [13]	*n*^a^; Covariates: none [13]
Subjective feelings regarding the earthquake	Correlation ratio with EPDS total score (ɳ=0.03, *p* > 0.05) [13]	*n*^a^; Covariates: none [13]
Sense of Coherence (SOC) total score	Correlation coefficient with EPDS total score (*r* = 0.21, *p* > 0.05) [13]	*n*^a^; Covariates: none [13]

Note: *CHQ* Chinese Health Questionnaire, *EPDS* Edinburgh Postnatal Depression Scale, *IES-R* Impact of Event Scale-Revised, *n* number of people contributing data, *RMB* Renminbi (Chinese currency), ɳ correlation ratio, *r* correlation coefficient, *OR* odds ratio, *aOR* adjusted odds ratio

- Adjusted statistics are the results of multivariate analyses; others are the results of bivariate analyses

^a^Sample varies from 82 to 99 for the variables reported by Hibino et al. [13]

#### Earthquake experiences

While all studies were conducted in an earthquake context, only four [13, 15, 18, 19] examined some aspect of earthquake experiences as a factor affecting CMDs during pregnancy (Table 2). Ren et al. [19] found that women who had ‘wounded relatives’ had a significantly and independently higher experiences of depressive symptoms (adjusted regression coefficient 0.22, *p* = 0.002). Qu et al. [15] used a study-specific ‘12 event self-assessment scale’ to measure earthquake experiences. They found that women who had higher earthquake experiences had significantly higher odds of experiencing PTSD (with ≥2 IES-R scale score) (aOR, 1.80, 95% CI, 1.43; 2.26, *p* < 0.001). However, there was no significant association between earthquake experiences and depressive symptoms [15].

#### Social support

Ren et al. [19] used a standardised tool which captured three dimensions of social support: subjective support, objective support and support use as a potential covariate of depressive symptoms during pregnancy. They found a significant negative correlation between subjective support (*r* = − 0.372; *p* = 0.01), objective support (*r* = − 0.324; *p* = 0.01) and support use (*r* = − 0.320; *p* = 0.01) and EPDS total score. Subjective support remained significant (adjusted regression coefficient − 0.25, *p* = 0.001) in multivariable analysis but the other two dimensions of social support were not significant [19]. Lau et al. [17] found a similar result when they used ‘the Interpersonal Support Evaluation List, a 40-item standardised scale to measure ‘perceived availability of functional social support’. They found that women who perceived support as poor were more likely to experience depressive symptoms (aOR, 3.07, 95% CI, 1.21; 7.78, *p* < 0.05) [17].

#### Socio-demographic factors

Three studies found a significant association of antenatal CMDs with at least one socio-demographic factor. Qu et al. [15] found in multivariable analyses that younger women (aged 18 to 25 years) were more likely to experience PTSD (aOR, 0.10, 95% CI, 0.03; 0.31, *p* < 0.001) and depressive symptoms (aOR, 0.42, 95% CI, 0.20; 0.90, *p* = 0.025) compared to older women (≥30 years). Lau et al. [17] also found that women aged ≤25 years (compared > 25 years) had a higher chance of experiencing depression in univariate analysis (OR, 2.46, 95% CI, 1.26; 4.81, *p* < 0.05); but the association was not significant in multivariable analysis. Other studies [13, 16, 18, 19] did not find a significant association between age and antenatal CMDs.

Qu et al. [15] found that compared to more highly educated women, women with lower education were at increased risk of experiencing PTSD (*p* < 0.001), but they did not find an association with depressive symptoms in bivariate analysis. The significance for PTSD also disappeared in multivariate analysis. Lau et al. [17] found that women with less than secondary education had higher odds of experiencing depression in univariate analysis (OR, 3.10, 95% CI, 1.62; 5.62, *p* < 0.01) but the association was not significant in multivariable analysis. Dong et al. [16], and Ren et al. [19] did not find any significant association of education of women with depressive symptoms.

Ren et al. [19] found that women who were farmers had higher EPDS mean scores (13.07 ± 5.93) compared to others (9.65 ± 5.03) (*p* = 0.001). In Lau et al. [17]‘s study, women who were unemployed or in part-time employment had higher odds of experiencing depressive symptoms in univariate analysis (OR, 2.35, 95% CI, 1.24; 4.45, *p* < 0.05); the association disappeared in multivariable analysis. Qu et al. [15] found that women who had low family income had a higher chance of experiencing PTSD in univariate analysis (*p* < 0.001), but the association did not remain significant in a multivariable analysis. They also did not find a significant association between family income with depressive symptoms. In a similar case, Lau et al. [17] found that women who had ‘one partner only’ as a main financial support in a family were more likely experiencing depressive symptoms in univariate analysis (OR, 3.35, 95% CI, 1.67; 6.71, *p* < 0.01); the association was not significant in a multivariable analysis.

#### Intimate partner relationship

While the quality of intimate partner relationship is consistently related to the mental health of pregnant women [3], only three studies [15–17] examined an aspect of intimate partner relationship as a covariate of antenatal CMDs. Dong et al. [16] found that marital satisfaction (*r* = − 0.30, *p* < 0.01), ‘thoughts and feelings regarding the marriage and one’s spouse’ (*r* = − 0.25, *p* < 0.01), and ‘agreement on relationship matter’ (r = − 0.30, *p* < 0.01) were significantly negatively correlated with EPDS scale score. However, the association did not remain significant in multivariable analysis. Lau et al. [17] found that women having ‘higher marital conflict’ had a higher chance of experiencing depressive symptoms in multivariable analysis (aOR, 3.60, 95% CI, 1.76; 7.20, *p* < 0.01). Qu et al. [15] found that ‘quality of women’s marital and family relationship’ was significantly negatively correlated with depression (*r* = − 0.18, *p* < 0.01) but the association was not significant with PTSD. They did not find any significant association with depressive symptoms or PTSD in multivariable analyses.

#### Reproductive factors

Five studies [13, 15–17, 19] examined aspects of reproductive health as covariates of antenatal CMDs. Two studies found a significant positive association between gestational age and depressive symptoms (ɳ = 0.21, *p* < 0.05) [13]; (ɳ = 0.181, *p* < 0.05) [19]. Similarly, two other studies found that pregnant women in the second or third trimester at the time of the study had a higher chance of experiencing depressive symptoms (*p* = 0.025) [15]; (*p* = 0.033) [19].

Two studies found that multiparous women were more likely experiencing depressive symptoms than nulliparous women (aOR, 2.47, 95% CI, 1.18; 5.17, *p* < 0.05) [17]; (adjusted regression coefficient = 0.262, *p* < 0.001) [19]. On the other hand, Qu et al. [15] did not find a significant association of multiparity with depressive symptoms. It was significantly associated with PTSD in univariate analyses (*p* < 0.001) but not in multivariable analyses.

Qu et al. [15] and Dong et al. [16] assessed ‘perceived pressure of pregnancy’ using an ‘11-item self-assessment scale’. They found that women who had higher perceived pressure were more likely experiencing PTSD (aOR, 1.19, 95% CI, 1.07; 1.32, *p* = 0.001) and depressive symptoms (aOR, 1.19, 95% CI, 1.12; 1.27, *p* < 0.001) [15]; (aOR, 4.55, 95% CI, 2.36; 8.77, *p* < 0.001) [16].

#### Personality

Ren et al. [19] examined different ‘coping styles’ as covariates of depressive symptoms. They found that a higher level of positive coping was protective against experiencing depressive symptoms (adjusted regression coefficient = − 0.193, *p* = 0.006). On the other hand, they found that a higher level of negative coping was a risk factor for depressive symptoms (adjusted regression coefficient 0.276, *p* < 0.001).

Dong et al. [16] measured life satisfaction as a covariate and found that it was significantly negatively correlated with depression (ɳ= − 0.25, *p* < 0.01). However, they did not find a significant association in multivariable analysis.

In summary, these studies reported that wounded relatives, poor social support, younger age, new residence of the study area, higher marital conflict, medium support from husbands, multiparity, the perceived stress of pregnancy, and negative coping were risks, and positive coping was protective against antenatal CMDs in multivariable analyses. It was also found that a higher composite score of earthquake experiences, younger age, and perceived stress of pregnancy were a risk factor for PTSD during pregnancy in multivariable analyses.

## Discussion

To our knowledge, this is the first systematic review of the evidence available about clinically-significant symptoms of antenatal CMDs among women who had recently experienced an earthquake. It provides a narrative synthesis of the existing evidence, but we acknowledge that because of the heterogeneity of these studies, we could not conduct a meta-analysis and provide meaningful estimates of the prevalence of pregnancy mental health problems among women who have experienced an earthquake. We also acknowledge that as the review was limited to the English-language literature, it is possible that articles published in languages other than English have been missed. Nevertheless, this review provides a comprehensive summary and evaluation of the available evidence in the field.

Only seven studies, all from high and middle-income countries met inclusion criteria for this review. They reported a wide range of prevalence of clinically-significant symptoms of antenatal CMDs. The differences in prevalence estimates might be attributable to study methods, but, as has been found in other post-disaster research, can reflect the local circumstances and post-disaster responses [20].

Standard study methods include the use of an adequately powered sample size, following standard sample recruitment strategies, and the use of valid and reliable outcome measures*.* The studies had varied sample size—small to relatively large—and none justified the sample size or representative adequacy of the samples. This could bring varied results of prevalence of antenatal CMDs. In addition, different measures of psychological symptoms were used, which reduces comparability and might account in part for the wide variation in prevalence estimates. Most importantly, only two studies [15, 16] used cut off scores that had been established in formal local validation studies among pregnant women. Without valid cut off scores, the prevalence estimates cannot be assumed to be accurate. It is not clear, therefore, how confidently the findings may be generalized.

There were differences in earthquake characteristics and settings which might also have influenced the outcomes. It is likely that women who were near the epicentres had more severe earthquake experiences compared to those up to 90 km away and they were more likely to experience a higher prevalence of antenatal CMDs. For instance, Lau et al. [17] reported 7.1%, and Ren et al. [19] reported 35.2% depressive symptoms using EPDS cut off score > 14 among women who experienced the earthquake during pregnancy. While Ren et al. [19] recruited pregnant women from the area of the epicentre of an earthquake scoring seven on the Richter Scale, Lau et al. [17] recruited pregnant women from 90 km away from the epicentre of an earthquake scoring eight on the Richter Scale.

The wide prevalence estimates of pregnancy mental health problems might also be attributable to local circumstances and post-disaster response. Compared to the prevalence reported from China, the prevalence in Japan was consistently lower. Japan is a well-resourced country with experience of earthquakes and well-developed post-disaster practices. These may assist more rapid recovery of post-adversities and explain why Japanese women appear to have a less negative psychological impact from experiencing an earthquake. In general circumstances too, Japanese have less prevalence of mental health problems compared to other high-income countries [21]. On the other hand, women in rural China may experience greater loss of close relatives, housing and livelihoods but may delay receiving support from significant others [19]. This may explain the higher prevalence in China. Nevertheless, the prevalence reported in Japan in an earthquake context was higher [13, 14] than those reported among non-earthquake affected pregnant women [22] in Japan. Usuda et al. [22] conducted a study among 177 pregnant women recruited from Toda Chuo Women’s Hospital (TCWH), Saitama Prefecture in the greater Tokyo area. The study reported that 1.1% of pregnant women met diagnostic criteria for major depression on The Mini-International Neuropsychiatric Interview (MINI) [22].

Dong et al.’s study [16] conducted four years after the Sichuan, China earthquake showed a high prevalence of depressive symptoms. However, the prevalence was not statistically significantly different to what has been reported from the sample of their comparison group that was recruited from a different location. To our knowledge, there is no study that conducted in the non-earthquake context in Sichuan, China to report the prevalence of antenatal CMDs. Nevertheless, Lee et al.’s study [23] that was conducted in general circumstances in an antenatal clinic of a regional hospital in Hong Kong, China found that pregnant women experienced a high prevalence of depressive symptoms: (22.1% (95% CI, 19.9; 24.4%) at first trimester, 18.9% (95% CI, 16.8; 21.1%) at second trimester, and (21.6% (95% CI, 19.4; 28.9%) at third trimester of pregnancy [23]. Even though Hong Kong is not directly comparable to Sichuan because of their socio-economic differences, it indicates that pregnant women in Sichuan experienced a higher prevalence of depressive symptoms in an earthquake context [15, 16] than the prevalence among pregnant women in Hong Kong [23]. It was reported that the Chinese government together with international agencies provided prompt support including psychological counselling immediately after the earthquake [24]. That support could ease the impact of the earthquake on pregnancy mental health. It can also be interpreted as the post-disaster effect on antenatal depressive symptoms may reduce over the time [16].

While these studies contribute to enhancing understanding of the field, they do not show clearly whether and how much earthquake experiences determine the mental health of pregnant women. Without a robust examination of potential factors together with earthquake experiences, it is not possible to conclude whether and how much earthquake experiences contributed to increasing mental health problems during pregnancy. However, this evidence is crucial for making public health decisions about whether to address post-earthquake antenatal mental health problems with universal or targeted strategies.

## Implications and conclusion

This systematic review found that women with recent direct experience of an earthquake appear to be at higher risk of clinically-significant antenatal CMD symptoms. These findings have implications for disaster responses. At present, the recommendation in this situation is that provisions for a safe birth and neonatal care [25] should be a priority. These data indicate that the mental health of women who are pregnant should also be considered and addressed. Pregnant women who reside in direct hit area or closer to the epicentre of the earthquake require special attention and support.

It also identifies knowledge gaps, in particular, that there is no evidence about the mental health of women who are pregnant and living in low-income nations at the time of an earthquake.

It is clearly a priority, that, despite the difficulties of conducting ethical, sensitive, comprehensive, culturally-competent research in such situations, it is needed in order to provide the evidence to inform effective interventions. At a minimum, this research should include standardised measures of earthquake experiences and examine all potential risk and protective factors in order to delineate the nature, prevalence and duration of antenatal mental health problems among women.
